# Diagnostic Performance of Clinical Examination for Detecting Knee Effusion: Impact of Obesity and Periarticular Adiposity

**DOI:** 10.7759/cureus.116265

**Published:** 2026-09-15

**Authors:** Naima Chakyr, Fatine Kronbi, Chaimae Nouri, Ayda Belmlih, Zhor Zeghari, Hanan Rkain, Latifa Tahiri, Fadoua Allali

**Affiliations:** 1 Department of Rheumatology B, Faculty of Medicine and Pharmacy, Ayachi Hospital, Ibn Sina Hospital Center, Mohammed V University, Rabat, MAR; 2 Department of Public Health, Laboratory of Community Health, Preventive Medicine, and Hygiene, Faculty of Medicine and Pharmacy, Mohammed V University, Rabat, MAR; 3 Laboratory of Biostatistics, Clinical Research, and Epidemiology, Faculty of Medicine and Pharmacy, Mohammed V University, Rabat, MAR; 4 Exercise Physiology and Autonomous Nervous System Team, Physiology Laboratory, Faculty of Medicine and Pharmacy, Mohammed V University, Rabat, MAR

**Keywords:** body mass index, knee effusion, patient perception, physical examination, ultrasonography

## Abstract

Introduction: Knee joint effusion is generally readily detected on clinical examination. However, the clinical examination may be negative despite ultrasonographic confirmation of an effusion. The aim of our study was to assess the diagnostic performance of clinical examination compared with that of ultrasonography for detecting knee effusion, identify factors associated with failure to detect an ultrasonographically confirmed effusion on clinical examination, and evaluate patients’ perception of knee swelling and the factors influencing this perception.

Methodology: A cross-sectional study was conducted in 200 patients (400 knees). Ultrasonography was considered the reference standard. Sensitivity, specificity, predictive values, diagnostic accuracy, and likelihood ratios were calculated. Factors associated with the outcomes of interest were analyzed using logistic regression based on generalized estimating equations (GEEs) to account for the correlation between the two knees of the same patient.

Results: Ultrasonographic evidence of knee effusion was present in 223 knees. Clinical examination had a sensitivity of 31.8%, a specificity of 95.5%, a positive predictive value of 89.9%, a negative predictive value of 52.6%, a diagnostic accuracy of 60.0%, a positive likelihood ratio of 7.04, and a negative likelihood ratio of 0.71. Sensitivity increased with ultrasonographic grade, from 29.6% for grade 1 to 33.8% for grade 2 and 40.9% for grade 3. In multivariable analysis, overweight, obesity, and local periarticular fat were associated with failure to detect effusion on clinical examination. Patients' perception of swelling had a sensitivity of 50.2%, a specificity of 69.5%, a positive likelihood ratio of 1.65, and a negative likelihood ratio of 0.72. Failure to perceive an effusion was associated with older age, educational level, and less severe pain, whereas in the absence of effusion, greater pain intensity was associated with more frequent perception of swelling.

Conclusions: Clinical examination had low sensitivity despite high specificity. The risk of non-detection was particularly high in patients with overweight or obesity and in those with greater periarticular fat, situations in which ultrasonography may be useful. Patients’ perception of swelling did not consistently correspond to the presence of an effusion and appeared to be strongly influenced by the visual analog scale for pain.

## Introduction

Knee joint effusion is a common manifestation of various rheumatic diseases, including osteoarthritis, rheumatoid arthritis [1,2], spondyloarthritis, and crystal-induced arthropathies. Its detection is essential for establishing the diagnosis, assessing disease activity, and guiding therapeutic management [1,3]. In clinical practice, clinical examination remains the first-line approach for detecting knee effusion [2,4,5]; however, its diagnostic performance may be influenced by patient characteristics and the extent of the effusion [1,3,4].

Musculoskeletal ultrasonography is recognized as a sensitive and reliable method for detecting knee joint effusion [1,3,6]. It not only confirms its presence but also allows assessment of its extent [3,6]. However, ultrasonographic findings may differ from those obtained on clinical examination by the physician [1,2]. Factors that may explain failure to detect an effusion on clinical examination, particularly the roles of body mass index (BMI) and periarticular fat, have been poorly investigated [7]. To our knowledge, data specifically concerning the Moroccan population remain limited.

The present study aimed to evaluate the diagnostic performance of clinical examination for detecting knee effusion, using ultrasonography as the reference standard; to identify factors associated with failure to detect an ultrasonographically confirmed knee effusion on clinical examination; and to assess patients’ perception of swelling and identify factors that may influence this perception.

## Materials and methods

Study design and population

This was an observational cross-sectional study conducted between July 20, 2026, and August 19, 2026, among patients presenting with knee pain to the Rheumatology B Department at Al Ayachi Hospital. Eligible patients were consecutively recruited during the predefined study period. No formal a priori sample size calculation was performed; the study included all eligible patients recruited during this period. The study included 200 patients, corresponding to the assessment of 400 knees. Patients aged 18 years or older who presented with knee pain were included. Patients were excluded if they had undergone recent knee surgery, had a neurological or sensory disorder affecting knee sensation, or had a cognitive disorder likely to impair the perception of swelling.

Data collection 

The sociodemographic and clinical data collected included age, sex, educational level, underlying disease, history of joint effusion, disease duration, BMI, and knee pain intensity assessed using a visual analog scale (VAS) for pain.

Patients were classified into four categories according to BMI, based on World Health Organization (WHO) cutoffs: underweight, BMI <18.5 kg/m²; normal weight, 18.5 kg/m² ≤ BMI ≤ 24.9 kg/m²; overweight, 25 kg/m² ≤ BMI ≤ 29.9 kg/m²; and obesity, BMI ≥ 30 kg/m² [8,9].

Clinical assessment of the knee

The clinical assessment of each knee was performed with the patient in the supine position. Knee effusion was assessed using the patellar tap test, performed by the examining rheumatologist. With the knee extended, the examiner compressed the suprapatellar pouch distally to displace intra-articular fluid toward the patellofemoral compartment and then applied downward pressure over the patella. The test was considered positive when a palpable tap or rebound of the patella against the femoral trochlea was elicited, indicating the presence of an effusion; in the absence of this finding, the test was considered negative.

Before the start of the study, a preliminary assessment was performed in 10 patients to evaluate the examiner’s ability to perform the clinical knee examination reproducibly. Intraobserver agreement was high, with a Cohen’s kappa coefficient of 0.80.

Skinfold thickness was measured on the medial aspect of the knee, 5 cm above the superior border of the patella, using a Harpenden Skinfold Caliper (Baty International, Unit 1 Sovereign Centre, West Sussex, United Kingdom).

Assessment of patients' perception

Subjective perception of knee swelling was assessed by direct questioning of the patient and coded as a binary variable (yes/no), according to the reported presence or absence of swelling or joint effusion.

This assessment reflected the patient’s subjective perception and was analyzed separately from the clinical examination.

Ultrasonographic assessment of the knee

The ultrasonographic examination was performed at the same time as the clinical examination by a rheumatologist other than the clinical examiner. Ultrasonography, used as the reference standard, was performed to confirm or exclude the presence of joint effusion [10,11].

Knee ultrasonography was performed in B-mode using a MyLab X6 ultrasound system equipped with a high-frequency SL2325 linear transducer (Esaote S.p.A., Genova, Italy), with the patient in the supine position and the knee flexed at 30°. Effusion was assessed in the suprapatellar recess as well as the medial and lateral recesses. Depth, gain, and focal settings were adjusted according to the patient’s body habitus to optimize visualization of the joint effusion.

Knee effusion was assessed ultrasonographically using a four-grade semiquantitative scale: grade 0, absence of effusion; grade 1, minimal effusion; grade 2, moderate effusion; and grade 3, marked effusion [11,12].

Local periarticular fat thickness was measured by ultrasonography on the medial aspect of the knee, 5 cm above the patella, and analyzed as a continuous quantitative variable.

The clinician performing the physical examination was blinded to the patient’s perception of swelling, while the ultrasonographer was blinded to the findings of the clinical examination [13].

Statistical analysis

Quantitative variables were expressed as mean ± standard deviation when normally distributed, or as median (interquartile range) otherwise. Qualitative variables were described as counts and percentages.

Ultrasonography was considered the reference standard. The diagnostic performance of clinical examination and patients’ perception of swelling was assessed across all 400 knees using sensitivity, specificity, positive and negative predictive values, diagnostic accuracy, and likelihood ratios, with their corresponding 95% confidence intervals (CIs) [13].

Factors associated with clinical non-detection, failure to perceive an existing effusion, and perception of swelling in the absence of effusion were investigated using logistic regression with generalized estimating equations (GEEs) to account for the correlation between the two knees of the same patient [14,15]. For each analysis, the outcome was modeled as a binary variable. A binomial distribution with a logit link function was specified, with patient identification used as the clustering variable. An exchangeable working correlation structure and a robust sandwich covariance estimator were used. Univariable analyses consisted of separate GEE models for each candidate predictor, followed by multivariable GEE models. Crude and adjusted odds ratios (ORs) with 95% CIs were reported. Two-sided Wald tests were used, and a p-value <0.05 was considered statistically significant. There were no missing data for the variables included in the analyses. For the analysis of clinical non-detection, age, BMI category, pain VAS score, and ultrasonographically measured periarticular fat thickness were included in the multivariable model. Age, pain VAS score, and periarticular fat thickness were entered as continuous variables, whereas BMI category was entered as a categorical variable, with normal weight as the reference category. The correlation between skinfold thickness and ultrasonographically measured periarticular fat thickness was assessed using Pearson’s correlation coefficient. Because these two measures were strongly correlated and assessed the same construct of local adiposity, they were not entered simultaneously into the multivariable model to reduce redundancy and potential multicollinearity; ultrasonographically measured periarticular fat thickness was retained. This analysis was restricted to knees with ultrasonographically confirmed effusion.

For the analyses of patients’ perception of swelling, age, educational level, disease duration, history of joint effusion, BMI category, pain VAS score, and ultrasonographically measured periarticular fat thickness were included in the multivariable GEE models. Age, disease duration, pain VAS score, and periarticular fat thickness were entered as continuous variables, whereas educational level, history of joint effusion, and BMI category were entered as categorical variables, with normal weight used as the reference category for BMI. Two separate outcomes were examined: failure to perceive swelling among knees with ultrasonographically confirmed effusion, and perception of swelling among knees with a negative ultrasonographic examination. The underweight category was excluded from the multivariable model for perception of swelling in the absence of effusion because of the very small number of patients in this category and the resulting instability of the estimates. The same GEE specifications were applied to both models.

Ethical considerations

The study was conducted in accordance with the Declaration of Helsinki and was approved by the Biomedical Research Ethics Committee of Mohammed V University, Faculty of Medicine and Pharmacy of Rabat and Faculty of Dental Medicine of Rabat, Morocco (approval number: 123/26).

Written informed consent was obtained from all patients before their inclusion in the study.

## Results

Characteristics of the study population

The demographic and clinical characteristics of the patients are summarized in Table 1. A total of 200 patients (400 knees) were included. The mean age was 57.6 ± 12.0 years. The study population was predominantly female, with 188 (94%) patients, and 137 (68.5%) patients had no formal education. The main etiologies identified were knee osteoarthritis in 126 (63%) patients, inflammatory rheumatic diseases in 67 (33.5%) patients, and metabolic rheumatic diseases in 7 (3.5%) patients. A history of knee effusion was reported in 128 (64%) patients, whereas 72 (36%) patients had no such history. The median disease duration was five years (IQR, 2-10). Regarding BMI categories, 88 (44%) patients had obesity, 69 (34.5%) were overweight, 40 (20%) had normal weight, and 3 (1.5%) were underweight.

**Table 1:** Demographic and clinical characteristics of the patients. *Data are presented as counts (percentages), except for age, presented as mean ± SD, and disease duration, presented as median (Q1-Q3). BMI, body mass index; SD, standard deviation; IQR, interquartile range

Variables	*N* = 200 (%)*
Age (years), mean ± SD	57.6 ± 12.0
Sex	
Female	188 (94)
Male	12 (6)
Educational level	
No formal education	137 (68.5)
Formal education	63 (31.5)
Underlying disease	
Inflammatory rheumatic diseases	67 (33.5)
Metabolic rheumatic diseases	7 (3.5)
Knee osteoarthritis	126 (63)
History of joint effusion	
Yes	128 (64)
No	72 (36)
Disease duration (years), median (IQR)	5 (2-10)
BMI category	
Underweight	3 (1.5)
Normal weight	40 (20)
Overweight	69 (34.5)
Obesity	88 (44)

Clinical and ultrasonographic characteristics of the knee are summarized in Table 2. Knee assessment showed a median skinfold thickness of 12 mm (IQR, 10-15), a mean local periarticular fat thickness of 13.9 ± 3.9 mm, and a median pain VAS score of 4 (IQR, 2-6).

**Table 2:** Clinical and ultrasonographic characteristics of the knee. VAS, visual analog scale; SD, standard deviation; IQR, interquartile range

Variables	*N* = 400
Skinfold thickness measured using a skinfold caliper (mm), median (IQR)	12 (10-15)
Periarticular fat thickness measured by ultrasonography (mm), mean ± SD	13.9 ± 3.9
Pain (VAS) score, median (IQR)	4 (2-6)

Diagnostic performance of clinical examination and patients’ perception of swelling compared with ultrasonography for the detection of knee effusion

Clinical examination had a sensitivity of 31.8% and a specificity of 95.5%, whereas patients' perception of swelling had a sensitivity of 50.2% and a specificity of 69.5%. Predictive values, diagnostic accuracy, and likelihood ratios are detailed in Table 3.

**Table 3:** Diagnostic performance measures of clinical examination and patients' perception. CI, confidence interval; LR+, positive likelihood ratio; LR-, negative likelihood ratio; PPV, positive predictive value; NPV, negative predictive value

Knee assessment method	Sensitivity (95% CI)	Specificity (95% CI)	PPV (95% CI)	NPV (95% CI)	Diagnostic accuracy (95% CI)	LR+ (95% CI)	LR- (95% CI)
Clinical examination	31.8% (25.8-38.4)	95.5% (91.3-98.0)	89.9% (81.0-95.5)	52.6% (47.0-58.2)	60.0% (55.0-64.8)	7.04 (3.49-14.24)	0.71 (0.65-0.79)
Perception of Swelling	50.2% (43.5-57.0)	69.5% (62.1-76.2)	67.5% (59.8-74.5)	52.6% (46.0-59.1)	58.8% (53.8-63.6)	1.65 (1.27-2.13)	0.72 (0.61-0.84)
Ultrasonographic Effusion Grade vs Clinical Examination							
Grade 1	29.6% (22.1-38.1)	95.5% (91.3-98.0)	83.3% (69.8-92.0)	64.2% (58.1-69.9)	67.1% (61.6-72.3)	6.59 (3.19-13.61)	0.73 (0.65-0.82)
Grade 2	33.8% (22.6-46.6)	95.5% (91.3-98.0)	73.3% (54.1-87.7)	79.8% (73.8-85.0)	79.0% (73.3-84.0)	7.53 (3.53-16.06)	0.69 (0.58-0.82)
Grade 3	40.9% (20.7-63.6)	95.5% (91.3-98.0)	52.9% (27.8-77.0)	92.9% (88.2-96.2)	89.5% (84.4-93.4)	9.10 (3.91-21.15)	0.61 (0.43-0.87)

The sensitivity of clinical examination increased with ultrasonographic grade, from 29.6% for grade 1 to 33.8% for grade 2 and 40.9% for grade 3. Specificity remained unchanged across all three grades at 95.5%. Predictive values and likelihood ratios are detailed in Table 3.

Factors associated with clinical nondetection of effusion in knees with ultrasonographically confirmed effusion

The results of the logistic regression analyses with GEE are summarized in Table 4. In univariable analysis, overweight, obesity, and periarticular fat thickness measured by ultrasonography were significantly associated with clinical nondetection of effusion. In multivariable analysis, overweight, obesity, and local periarticular fat thickness remained independently associated with clinical nondetection of effusion. Age, underweight, and pain VAS score were not significantly associated after adjustment.

**Table 4:** Factors associated with clinical nondetection of effusion: univariate and multivariable analyses. BMI, body mass index; VAS, visual analog scale; CI, confidence interval

Variables	Univariate analysis			Multivariable analysis		
	Odds ratio	95% CI	*P*-value	Odds ratio	95% CI	*P*-value
Age	0.822	0.64-1.05	0.125	0.834	0.61-1.12	0.236
BMI category						
Normal weight	Ref					
Overweight	6.639	2.43-18.13	<0.001	6.159	1.94-19.48	0.002
Obesity	15.722	5.53-44.62	<0.001	9.820	2.92-32.96	<0.001
Underweight	1.728	0.09-31.87	0.713	4.129	0.26-63.80	0.310
Pain VAS score	1.009	0.87-1.16	0.906	0.991	0.84-1.16	0.914
Periarticular fat thickness measured by ultrasonography	3.206	1.94-5.29	<0.001	2.220	1.26-3.88	0.005
Skinfold thickness measured using a skinfold caliper	1.200	1.09-1.31	<0.001			

Factors associated with failure to perceive swelling in knees with ultrasonographically confirmed effusion

The results of the logistic regression analyses with GEE are summarized in Table 5. In univariable analysis, older age was associated with higher odds of failure to perceive effusion. Conversely, a history of knee effusion was associated with lower odds of non-perception, as was a higher VAS for pain. In multivariable analysis, older age, having formal education, a history of knee effusion, and pain VAS score remained independently associated with failure to perceive effusion. 

**Table 5:** Factors associated with failure to perceive swelling: univariate and multivariable analyses. BMI, body mass index; VAS, visual analog scale; CI, confidence interval

Variables	Univariate analysis			Multivariable analysis		
	Odds ratio	95% CI	*P*-value	Odds ratio	95% CI	*P*-value
Age	1.047	1.01-1.07	0.004	1.056	1.02-1.09	0.002
Educational level	1.616	0.80-3.24	0.178	3.480	1.33-9.10	0.011
Disease duration	0.974	0.93-1.02	0.261	0.999	0.94-1.05	0.986
History of joint effusion	0.336	0.16-0.68	0.003	0.416	0.18-0.95	0.038
BMI category						
Normal weight	Ref					
Underweight	0.998	0.05-18.32	0.999	1.123	0.07-18.10	0.935
Overweight	1.433	0.51-3.98	0.491	1.225	0.35-4.19	0.747
Obesity	1.250	0.46-3.36	0.659	1.110	0.31-3.94	0.872
Pain VAS score	0.706	0.60-0.82	<0.001	0.693	0.57-0.83	<0.001
Periarticular fat thickness measured by ultrasonography	0.968	0.88-1.05	0.458	0.996	0.88-1.12	0.954

Factors associated with the perception of swelling in the absence of ultrasonographically confirmed effusion

The results of the logistic regression analyses with GEE are summarized in Table 6. In univariable analysis, educational level, a history of knee effusion, and pain intensity were associated with the perception of swelling. In multivariable analysis, after adjustment for age, educational level, disease duration, history of knee effusion, BMI category, VAS for pain, and ultrasonographically measured local periarticular fat thickness, only the pain VAS score remained significantly associated with the perception of swelling in the absence of effusion.

**Table 6:** Factors associated with the perception of swelling: univariate and multivariable analyses. The underweight category was not included in the multivariable model because of the small number of patients in this category and the resulting instability of the estimates. BMI, body mass index; VAS, visual analog scale; CI, confidence interval

Variables	Univariate analysis			Multivariable analysis		
	Odds ratio	95% CI	*P*-value	Odds ratio	95% CI	*P*-value
Age	1.014	0.98-1.04	0.353	1.007	0.96-1.04	0.710
Educational level	0.378	0.15-0.90	0.028	0.389	0.14-1.01	0.055
Disease duration	1.005	0.95-1.06	0.860	0.973	0.90-1.04	0.451
History of joint effusion	3.670	1.57-8.54	0.003	2.520	0.94-6.70	0.064
BMI category						
Normal weight	Ref					
Overweight	2.31	0.77-6.90	0.133	2.705	0.79-9.25	0.113
Obesity	1.95	0.70-5.37	0.196	1.666	0.49-5.63	0.412
Pain VAS score	1.454	1.21-1.73	<0.001	1.516	1.25-1.83	<0.001
Periarticular fat thickness measured by ultrasonography	1.076	0.98-1.17	0.090	1.081	0.97-1.20	0.154

## Discussion

The results of this study showed that clinical examination had low sensitivity but high specificity for detecting knee effusion, with ultrasonography considered the reference standard. A positive clinical examination was therefore strongly suggestive of the presence of an effusion. This confirmatory value was further supported by a high positive likelihood ratio, indicating that a positive clinical examination markedly increased the probability of an effusion being present. Conversely, the low sensitivity and the insufficiently low negative likelihood ratio limited the ability of a negative clinical examination to rule out an effusion, with a substantial number of cases detected only by ultrasonography.

The low sensitivity observed in our study is consistent with the literature, which consistently shows that ultrasonography is more sensitive than clinical examination for detecting knee effusions. In this regard, Kane et al. demonstrated in patients with rheumatoid arthritis that clinical examination underestimated the frequency of joint effusions, as well as other synovial abnormalities, compared with ultrasonographic assessment [1].

Our findings differ from those reported by Elsawy et al., who found, in a cohort of 109 knees with osteoarthritis, high sensitivity for visual inspection (90.5%) and the patellar tap test (86.5%), whereas the bulge sign showed more moderate sensitivity (63.5%) for detecting joint effusion [4]. In contrast, their specificities were lower, at 37.1%, 31.4%, and 65.7%, respectively [4]. This discrepancy may be explained by the fact that their study population consisted of patients selected for knee osteoarthritis with clinical or ultrasonographic suspicion of effusion, thereby increasing the proportion of more readily detectable effusions. It may also be related to the criteria used to define a positive clinical examination: their study assessed visual inspection, the patellar tap test, and the bulge sign separately, whereas our study relied on an overall clinical classification.

The meta-analysis by Meyer et al. also concluded that ultrasonography was more sensitive than the bulge sign and the patellar tap test for detecting knee effusion, with better ability to correctly identify both positive and negative cases [3]. These findings support our observation that the absence of effusion on clinical examination is insufficient to rule out its presence on ultrasonography.

The sensitivity of clinical examination increased with ultrasonographic grade. This trend is consistent with the concept that larger effusions are more readily detected on physical examination. A pilot study evaluating effusion volume showed that a positive patellar tap test or bulge sign was moderately predictive of a larger fluid volume [16]. However, in our population, even grade 3 effusions frequently remained undetected, suggesting that effusion size is not the only determinant of clinical detection.

This difficulty in detecting effusion may also be influenced by knee morphology. In our multivariable analysis, overweight, obesity, and periarticular fat thickness were associated with clinical non-detection of effusion. These findings suggest that greater adiposity may obscure peripatellar anatomical landmarks and reduce the performance of inspection and palpation maneuvers. However, they differ from those reported by Ulaşlı et al., who found no significant effect of obesity on the clinical detection of effusion [7]. This discrepancy may be related to differences in study populations, the distribution of BMI categories, and effusion severity. It may also be explained by the fact that, in addition to overall BMI, our study assessed local periarticular fat thickness, which may more directly affect the visibility and palpability of knee anatomical landmarks [7].

Regarding the subjective perception of swelling, its diagnostic performance compared with ultrasonography was limited, indicating a limited ability to correctly identify an effusion. These findings differ from those of MacFarlane et al., who reported, in patients with knee osteoarthritis and concomitant meniscal injury, higher sensitivity but lower specificity of patient-reported swelling compared with MRI-defined effusion [17]. This difference may be explained by the imaging methods and thresholds used: their study grouped absent and minimal effusions together and primarily considered moderate-to-large effusions as positive, whereas our study considered any ultrasonographic effusion graded 1 to 3 as present, including small effusions, which are likely more difficult for patients to perceive. Overall, both studies indicate that patient-reported swelling is not a reliable substitute for imaging to objectively confirm the presence of an effusion.

In our study, a higher pain VAS score was associated with better perception of swelling when an effusion was present, but also with more frequent perception of swelling when ultrasonography was negative. This finding suggests that pain may increase attention to the knee and lead patients to interpret sensations of tightness, stiffness, or discomfort as swelling. Conversely, a minimally painful effusion may go unnoticed. This dissociation between pain and effusion is consistent with evidence from the literature showing that painful symptoms are not always directly correlated with fluid volume or the presence of ultrasonographically detected effusion [18,19].

Educational level was also associated with failure to perceive effusion, with patients who had formal education having higher odds of failing to perceive swelling than those with no formal education. To our knowledge, studies specifically examining the association between educational level and perception of knee swelling are scarce. This finding should therefore be considered exploratory. It should also be interpreted with caution because patients with formal education were less numerous than those with no formal education in our sample, which may reduce the precision of the estimate. This association may reflect differences in how patients describe symptoms, distinguish pain from swelling, or interpret joint-related sensations, rather than a direct effect of educational level.

This study has several limitations. First, its single-center design may limit the generalizability of the findings to other populations. Clinical assessment was performed by a single examiner, and no interobserver reliability analysis was conducted. In addition, no a priori sample size calculation was performed, which may have limited the statistical power of some analyses and the precision of the estimates. The small sample size of certain subgroups, particularly men, underweight patients, and patients with grade 3 effusions, may also have reduced the precision of the estimates. Finally, the cross-sectional design of the study precludes establishing causal relationships between the identified factors and clinical non-detection, failure to perceive an existing effusion, or perception of swelling in the absence of ultrasonographically confirmed effusion.

## Conclusions

Although clinical examination showed good specificity, its sensitivity remains insufficient for the reliable detection of knee effusions, particularly in patients who are overweight, have obesity, or have increased periarticular adipose tissue. Patient-reported perception of swelling also showed limited discriminatory ability. Pain intensity was associated with patients’ perception of swelling, including in the absence of ultrasonographically confirmed effusion.

In this context, ultrasonography may serve as a complementary diagnostic tool, when clinical findings are equivocal or when clinical suspicion persists despite a negative physical examination.
